# Long-Distance Communication between Laryngeal Carcinoma Cells

**DOI:** 10.1371/journal.pone.0099196

**Published:** 2014-06-19

**Authors:** Ieva Antanavičiūtė, Kristina Rysevaitė, Vykintas Liutkevičius, Alina Marandykina, Lina Rimkutė, Renata Sveikatienė, Virgilijus Uloza, Vytenis Arvydas Skeberdis

**Affiliations:** 1 Institute of Cardiology, Lithuanian University of Health Sciences, Kaunas, Lithuania; 2 Institute of Anatomy, Lithuanian University of Health Sciences, Kaunas, Lithuania; 3 Department of Otorhinolaryngology, Lithuanian University of Health Sciences, Kaunas, Lithuania; Albert Einstein College of Medicine, United States of America

## Abstract

Tunneling nanotubes and epithelial bridges are recently discovered new forms of intercellular communication between remote cells allowing their electrical synchronization, transfer of second messengers and even membrane vesicles and organelles. In the present study, we demonstrate for the first time in primary cell cultures prepared from human laryngeal squamous cell carcinoma (LSCC) samples that these cells communicate with each other over long distances (up to 1 mm) through membranous tunneling tubes (TTs), which can be open-ended or contain functional gap junctions formed of connexin 43. We found two types of TTs, containing F-actin alone or F-actin and α-tubulin. In the LSCC cell culture, we identified 5 modes of TT formation and performed quantitative assessment of their electrical properties and permeability to fluorescent dyes of different molecular weight and charge. We show that TTs, containing F-actin and α-tubulin, transport mitochondria and accommodate small DAPI-positive vesicles suggesting possible transfer of genetic material through TTs. We confirmed this possibility by demonstrating that even TTs, containing gap junctions, were capable of transmitting double-stranded small interfering RNA. To support the idea that the phenomenon of TTs is not only typical of cell cultures, we have examined microsections of samples obtained from human LSCC tissues and identified intercellular structures similar to those found in the primary LSCC cell culture.

## Introduction

Physiological and pathological processes such as homeostasis, embryogenesis, development, tumorigenesis, and cell movement depend on the synchronization of cell-to-cell communication. Intercellular communication between cells is performed by soluble molecules of endocrine and paracrine signaling systems and by direct noncytoplasmic and cytoplasmic connections. Noncytoplasmic connections include cytonemes described in *Drosophila melanogaster* and some other invertebrate cells [1], [2] and filopodial bridges (viral cytonemes) found in mammalian cells [3], [4]. Cytonemes extend up to 100 µm and connect the anterior and posterior compartments of the imaginal disc in fruit flies. Similar structures have been reported in human neutrophils [5]. Filopodial bridges are shorter than 10 µm and can transfer retrovirus infection. In both cases, these membranous tubes contact the substratum and transfer cargoes along their outer surface. Cytoplasmic connections between contiguous cells can be achieved through plasmodesmata in plants [6] and gap junctions (GJs) in animals [7], [8]. Plasmodesmata are microscopic channels traversing cell walls that enable the transport of substances between cells. GJ channels are formed by 2 apposing hemichannels (aHC) (each composed of 6 connexin (Cx) subunits) and provide a direct pathway for electrical and metabolic signaling between adjacent cells. Cytoplasmic connections between remote cells have recently been discovered in cultured rat pheochromocytoma PC12 cells [9] and designated tunneling nanotubes (TNTs) (reviewed in refs. [10], [11]). These F-actin-based membranous structures, depending on the cell type, range from 20 to 800 nm in diameter and extend up to several cell diameters. They do not touch the substratum and have life times from minutes up to several hours.

The mechanism of TNT formation has not been completely elucidated yet. Two models of TNT formation have been proposed. The first model is based on the outgrowth of filopodium-like protrusions that elongate by F-actin polymerization and make physical contact with a remote cell establishing either an open-ended connection through membrane fusion or electrical coupling through GJs, or close-ended connections where the cargo has to traverse the plasma membrane boundary. LST1, a transmembrane MHC class III protein, is responsible for the formation of functional TNTs by recruiting filamin, an actin-crosslinking protein, to the plasma membrane and interacting with M-Sec, myosin and myoferlin [12]. The M-Sec protein was previously reported to be a central factor for F-actin polymerization-based TNT formation [13]. The second model is based on cell dislodgment after tight cell-cell contacts. Cells moving in opposite directions pull out the open-ended TNT that may rupture preserving tips in contact and establishing close-ended or GJ-based connections. Since their discovery in 2004, TNTs have been described in many other cell types where they have been shown to be implicated in the intercellular electrical coupling and Ca^2+^ flux; transfer of organelles or proteins; virus, pathogenic prion, and protein transmission; cell migration; and bacteria capture (reviewed in refs. [10], [11], [14], [15]). Interestingly, it has been shown in certain cell cultures that TNTs, in addition to F-actin, contain microtubules, and while cargo transport in solely F-actin containing TNTs is unidirectional, in microtubules containing TNTs, it is bidirectional [16].

More recently, 2 novel long-distance tubular channels between human bronchial epithelial cell islands and A549 human alveolar basal carcinoma cells have been discovered [17], [18]. Termed epithelial bridges (EPB1 and EPB2), these intercellular TTs differ structurally from TNTs. They range from 1 to 20 µm in diameter and provide direct intercellular communication over the longest distances (>1 mm) reported to date. All EPBs are F-actin and microtubule composites and, similar to TNTs, hover above the substratum. EPB1s, like TNTs and other intercellular channels, facilitate cellular material transport. In contrast, EPB2s provide conduits for a whole cell or cell groups to move from one epithelial cell island to another, representing a completely new mechanism of cell migration.

The first evidences on the existence of TNTs in tissues were presented in animals by Chinnery and colleagues in the mouse corneal stroma [19] and in humans by Lou and colleagues [20] in the pleural mesothelioma and adenocarcinoma specimens. Also, one recent publication has demonstrated TNT-like structures between migrating cells of the cultured explants from the metastatic nodules of ovarian cancer [21]. However, to our knowledge, there are no data in the literature about the presence of TNTs in other types of malignant tumors, including squamous cell carcinoma of the head and neck region.

In this study, we found that laryngeal squamous cell carcinoma (LSCC) cells in the culture performed electrical and metabolic communication via membranous TTs of nano- and microscale, similar to TNTs and EPBs, respectively. The thickest and longest intercellular tubes formed during cytokinesis provide open-ended connections, capable of transmitting at least 3-kDa molecules and transporting mitochondria. The TTs formed by the filopodium or lamellipodium outgrowth mechanism establish intercellular connections through Cx43-based GJ formation with consequent voltage gating properties and permeability for smaller molecules (<1.2 kDa). Moreover, we show for the first time that open-ended and even GJ-containing TTs can transfer the genetic material, such as siRNA. Finally, we demonstrate that TTs, containing F-actin alone and together with α-tubulin, exist in the LSCC tissues.

## Materials and Methods

### Human Laryngeal Carcinoma Cells and Tissues

Our investigations were performed in accordance with the principles outlined in the Declaration of Helsinki and approved by Kaunas Regional Bioethics Committee (BE-2-34, 2007). Histologically confirmed LSCC tissue samples were collected in accordance with the protocol approved by the Institutional Review Board, Lithuanian University of Health Sciences (LUHS). Informed written consent was obtained before surgery, and patient identifiers were removed to ensure anonymity.

LSCC tissue samples were obtained from the Department of Otorhinolaryngology, LUHS. The primary culture of LSCC cells was prepared from part of the tumor tissue sample taken from a 56-year-old male patient with T1a Nx Ro G2 LSCC during hemilaryngectomy surgery. The second part of the sample was used for a histological and immunohistochemical examination. LSCC staging, T (tumor) and N (node) categories were assigned according to the Union for International Cancer Control classification. The diagnosis was pathohistologically confirmed at the Department of Pathology, LUHS. The moderate differentiation grade (G2) of carcinoma was determined according to the Union for International Cancer Control classification. In addition, the specimens from 5 other patients with LSCC diagnosis were examined histologically and immunohistochemically.

The carcinoma tissue sample (∼0.5 cm^3^) was cut with scissors into smaller pieces, treated with 1-mg/mL collagenase (type V) and 0.125% trypsin in PBS, and shaken at 37°C for 2 h at 350 rpm. After washing with DMEM, the LSCC cells were seeded into flasks with a growth medium (DMEM, 10% FBS, penicillin 100 U/mL and streptomycin 100 µg/mL) and incubated at 37°C in a humidified atmosphere of 5% CO_2_. After 24 h, all dead and unattached cells were removed. The growth medium was changed every day until the adherent cells reached 90% confluence, and then they were re-seeded in new culture flasks. All chemicals were purchased from Sigma-Aldrich (Steinheim, Germany).

### Time-lapse Imaging

Time-lapse imaging of LSCC cell motility and TT formation in the culture medium was performed at 37°C in a humidified atmosphere of 5% CO_2_ using an incubation system INUBG2E-ONICS (Tokai Hit, Shizuoka-ken, Japan) with an incubator, mounted on the stage of the motorized Olympus IX81 microscope (Olympus Europe holding Gmbh, Hamburg, Germany) equipped with UPLFLN 4x/0.13, UPLFLN 10x/0.3, UPlanSApo 20x/0.85 OI, or PlanApo N 60x/1.42 OI lens, the Orca-R^2^ cooled digital camera (Hamamatsu Photonics K.K., Japan) and the fluorescence imaging system XCELLENCE (Olympus Soft Imaging Solutions GmbH, München, Germany). Differential interference contrast (DIC) or phase-contrast images were taken in addition to time-lapse imaging.

### Electrophysiological Measurements

For simultaneous electrophysiological and fluorescence recordings, the cells grown onto glass coverslips were transferred to an experimental chamber with constant flow-through perfusion mounted on the stage of the inverted microscope Olympus IX81 equipped with the Orca-R^2^ cooled digital camera, fluorescence excitation system MT10 (Olympus Life Science Europa Gmbh, Hamburg, Germany), and fluorescence imaging system XCELLENCE. The UPlanSApo 20x/0.85 OI lens and appropriate excitation and emission filters (Chroma Technology, Brattleboro, VT, USA) (all used filters are specified in **Table S1**) were used to image (molecular mass of the fluorescent ion; valence) Alexa Fluor-350 (AF350; 326; −1), Lucifer Yellow (LY; 443; −2), DAPI (279; +2), Alexa Fluor-488/3000 dextran (AF488/3000; 3000; −1), and siRNA conjugated with AF488 (siRNA/AF488; ∼15000; n.d.). Junctional conductance g_T_ between the cells connected by the TT was measured using the dual whole-cell patch-clamp technique. Cell-1 and cell-2 of a cell pair were voltage clamped independently with the patch-clamp amplifier MultiClamp 700B (Molecular Devices, Inc., USA) at the same holding potential, V_1_ = V_2_. Voltages and currents were digitized using the Digidata 1440A data acquisition system (Molecular Devices, Inc., USA) and acquired and analyzed using pClamp 10 software (Molecular Devices, Inc., USA). By stepping the voltage in the cell-1 (ΔV_1_) and keeping the other constant, junctional current was measured as the change in current in the unstepped cell-2, I_T_ = ΔI_2_. Thus, g_T_ was obtained from the ratio −I_T_/ΔV_1_, where ΔV_1_ is equal to transjunctional voltage (V_T_), and a negative sign indicates that the junctional current measured in the cell-2 is oppositely oriented to the one measured in the cell-1. To minimize the effect of series resistance on the measurements of g_T_ [22], we maintained pipette resistances below 3 MOhms. Patch pipettes were pulled from borosilicate glass capillary tubes with filaments. Experiments were performed at room temperature in a modified Krebs-Ringer solution (in mM): NaCl, 140; KCl, 4; CaCl_2_, 2; MgCl_2_, 1; glucose, 5; pyruvate, 2; HEPES, 5 (pH 7.4). Patch pipettes were filled with saline containing (in mM): KCl, 130; Na aspartate, 10; MgATP, 3; MgCl_2_, 1; CaCl_2_, 0.2; EGTA, 2; HEPES, 5 (pH = 7.3). AF350 and AF488/3000 were purchased from Invitrogen; AllStars negative control siRNA/AF488 was obtained from Qiagen. All other chemical reagents were purchased from Sigma-Aldrich Corp.

### Fluorescence Imaging and Dye Transfer Studies

Fluorescence signals were acquired using the Olympus IX81 microscope with UPlanSApo 20x/0.85 OI lens, Orca-R^2^ digital camera, fluorescence excitation system MT10, and XCELLENCE software. For dye transfer studies, a given dye was introduced into the cell-1 of a pair through a patch pipette in the whole-cell voltage-clamp mode. Typically, this resulted in the rapid loading of the cell-1, followed by dye transfer via the TT to the neighboring cell-2. A whole-cell recording in the dye recipient cell (cell-2) was established ∼10–30 min after opening the patch in the cell-1. This allowed measurement of g_T_ and avoided dye leakage into the pipette-2 during the measurements of dye permeability. Evaluation of GJ permeability to dyes from changes in fluorescence intensity in both cells was previously described elsewhere [23]–[25]. In brief, the cell-to-cell flux (J_T_) of the dye in the absence of transjunctional voltage (V_T_ = 0 mV) can be determined from the changes of dye concentration in the cell-2 (ΔC_2_) over the time interval (Δt) as follows:
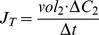
(1)where vol_2_ is the volume of cell-2. Then, according to the modified [26] Goldman-Hodgkin-Katz (GHK) equation [27], the total junctional permeability (P_T_) can be described in consequence:
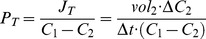
(2)where C_1_ and C_2_ are dye concentrations in the cell-1 (dye donor) and the cell-2 (dye recipient), respectively. Cell volume was approximated as a section of a sphere and calculated from the formula vol_2_ = (1/6) · π · h · (3a^2^+ h^2^). The diameter of the base (a) was determined by averaging the longest and shortest diameters of the cell; the section height (h) was measured by XCELLENCE software changing a focus from the base to the top of the cell. On average, the volume of examined LSCC cells was ∼68,000 µm^3^. Assuming that the dye concentration is directly proportional to fluorescence intensity (C = k · FI), the equation 2 can be modified as follows:
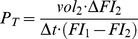
(3)where ΔFI_2_ = FI_2,n+1_– FI_2,n_ is the change in FI in the cell-2 over time, Δt = (t_n+1_– t_n_); n is the nth time point in the recording. Most of the fluorescent dyes and reagents were purchased from Invitrogen (Eugene, OR, USA). To minimize dye bleaching, studies were performed using time-lapse imaging, which exposed cells to low-intensity light for ∼0.5 s every 1 min. We also used dyes at low concentrations in the pipette solution, typically 0.1 mM and below, which minimized phototoxicity but still provided satisfactory fluorescence intensities.

### Histology

For a histological visualization, part of each freshly dissected tissue sample was fixed for 3 days at 4°C in a 4% paraformaldehyde solution in 0.01 M phosphate buffer (pH 7.4), routinely embedded in paraffin, and sectioned in 4–5-µm thick slices employing a rotatory microtome Microm HM350S (Germany). Tissue sections were stuck on microscope slides by electrostatic attraction (SuperFrost Ultra Plius, Thermo Scientific, Braunschweig, Germany) and dried up to 12 h at 50°C. Staining with Masson’s trichrome (Sigma-Aldrich, Germany) and hematoxylin-eosin (Sigma-Aldrich, Germany) was used to characterize LSCC. The preparations were examined with the fluorescent microscope AxioImage M1 (Zeiss, Gottingen, Germany). Bright field images were taken using EC Plan-Neofluar 10x/0.3, Plan-Apochromat 20x/0.8, EC Plan-Neofluar 40x/0.75, or Plan-Apochromat 63x/1.40 OI lens and the high-resolution color camera AxioCam HRc (Zeiss, Gottingen, Germany).

### Immunohistochemistry of Cells and Tissues

#### Cell culture

Cells were grown in 24-well plates with glass coverslips on the bottom, fixed with 4% paraformaldehyde for 15 min, and permeabilized with 0.2% Triton X-100/PBS for 3 min. Coverslips were incubated for 1 h with the following primary antibodies: mouse anti-α-tubulin (T5168, Sigma-Aldrich, Steinheim, Germany), rabbit anti-Cx43 (710700, Invitrogen, USA), mouse anti-Cx43 (610061, Transduction Laboratories, Lexington, KY, USA), rabbit anti-Cx26 (71-0500, Invitrogen, USA), rabbit anti-Cx30 (HPA014846, Sigma-Aldrich, Steinheim, Germany), then rinsed with 1% BSA/PBS and incubated with secondary goat anti-mouse IgG H&L (Cy5) (ab6563, Abcam Cambridge, UK) or with donkey anti-rabbit IgG (FITC) (sc-2090, Santa Cruz, CA, USA) for 30 min. The F-actin network was visualized using Alexa Fluor 594 phalloidin (Invitrogen, USA); coverslips were incubated with the dye for 30 min at 37°C. Coverglasses were attached using Vectashield Mounting Medium with DAPI (Vector Laboratories, CA, USA) and sealed with clear nail polish. MitoTracker Green (Invitrogen, USA) was used to stain mitochondria in live cells following the manufacturer’s instructions. Analysis was performed using the Olympus IX81 microscope with UPlanSApo 20x/0.85 OI or PlanApo N 60x/1.42 OI lens and the Orca-R^2^ digital camera with the fluorescence excitation system MT10 and XCELLENCE software.

#### Tissues

Freshly dissected tissues were immersed in 4% paraformaldehyde in PBS for 24 hours at 4°C, then transferred to 20% sucrose in PBS for 24 at 4°C, and frozen on specimen plates by using a TBS tissue freezing medium (Triangle Biomedical Sciences, Durham, NC, USA). Tissue samples were sectioned at a thickness of 25 µm in a microtome cryostat (Microm HM560M, Germany) at −20°C. Sections were collected on SuperFrost slides and air dried for 30 min at room temperature. Slide-mounted tissue sections were washed in PBS, permeabilized with 0.5% Triton X-100 in PBS containing 0.5% BSA, and blocked for 1 h in PBS containing 5% normal donkey serum (Jackson Immunoresearch Laboratories, West Grove, PA, USA). Tissues were then incubated for 1 h with mouse anti-α-tubulin (T5168, Sigma-Aldrich, Steinheim, Germany) or rabbit anti-Cx43 (710700, Invitrogen, USA), rabbit anti-Cx26 (71-0500, Invitrogen, USA), and rabbit anti-Cx30 (HPA014846, Sigma-Aldrich, Steinheim, Germany) primary antibodies for 18–20 hours at 4°C in a humid chamber. Tissues were then washed with PBS and incubated for 2 h with species-specific donkey anti-mouse or donkey anti-rabbit secondary antibody conjugated with FITC (AP192F or AP182F, respectively, Chemicon, California, USA). After washing in PBS, the actin network was visualized using Alexa Fluor 594 phalloidin (Invitrogen, USA). Mitochondria were stained with an anti-mitochondria [MTC02] antibody (ab3298, Abcam, Cambridge, UK) and a donkey anti-mouse secondary antibody conjugated with FITC (AP192F, Chemicon, California, USA). Coverglasses were attached using Vectashield Mounting Medium with DAPI (Vector Laboratories, CA, USA) and sealed with clear nail polish. Both positive and negative controls were used. The preparations were examined by the fluorescent microscope AxioImage Z1 fitted with Apotome and fluorescence filter sets No. 38HE, 43HE, and 49 (Karl Zeiss, Gottingen, Germany), and with Plan-Apochromat 20x/0.8, EC Plan-Neofluar 40x/0.75, and Plan-Apochromat 63x/1.40 OI lenses (Karl Zeiss, Gottingen, Germany). Fluorescent images were taken using the monochrome camera AxioCam MRm (Zeiss, Gottingen, Germany). The optically sectioned stacks of images were projected into the final image using a special extended focus module available in AxioVision (v. 4.8.2, Karl Zeiss, Gottingen, Germany) software. When necessary, the same preparations were additionally analyzed and photographed employing the laser scanning microscope LSM 700 (Zeiss, Jena, Germany) with its ZEN 2010 software (Zeiss, Jena, Germany).

### Data Analysis and Statistics

The analysis was performed using SigmaPlot software (Systat, Richmond, CA, USA), and averaged data are reported as means ± SEM.

## Results

### General Properties of LSCC Tissue and Primary Cell Culture

**Figure 1:**
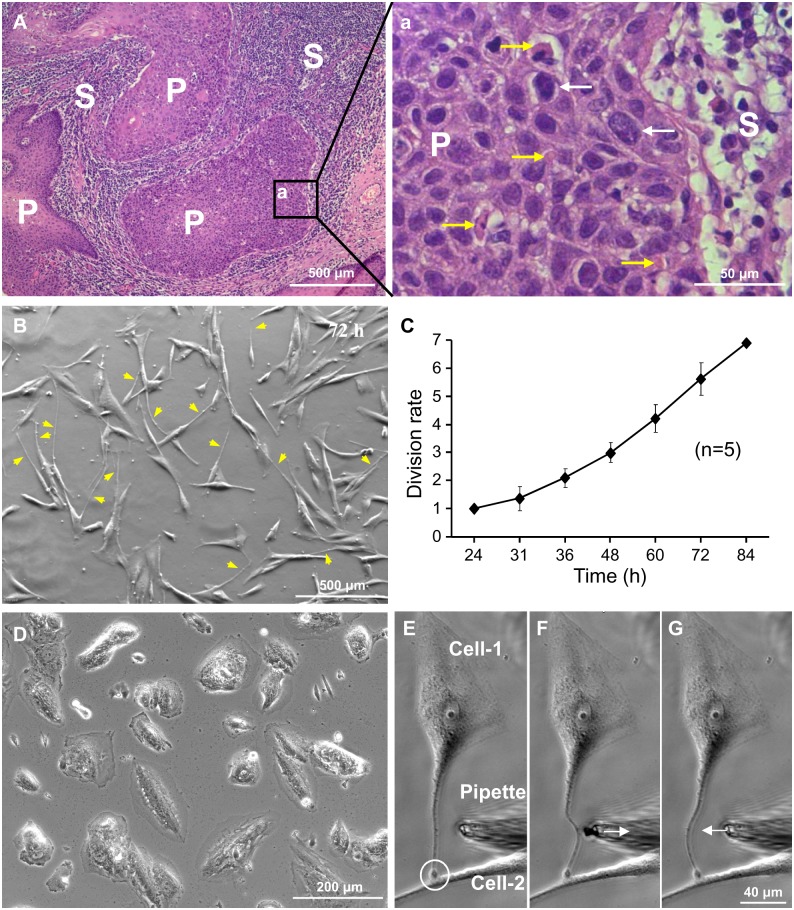
Characterization of LSCC cells. (A and a) Moderately differentiated squamous cell carcinoma of the larynx. The parenchyma (P) of the tumor consists of atypical epithelial cells with numerous hyperchromatic giant nuclei and atypical mitoses (white arrows). Several keratinizing cells are shown by yellow arrows. The surrounding stroma (S) is composed of the connective tissue with lymphocyte infiltration (hematoxylin and eosin staining). (B) LSCC cells after 3 days *in vitro* (TTs are indicated by yellow arrows). (C) Division rate of LSCC cells at the eighth passage (n = 5). No major differences in the division rate were observed between passages 5 to 15. (D) Preincubation of LSCC cells with colchicine (10 µM), an inhibitor of microtubule polymerization, for 24 h resulted in a complete loss of TTs. (E–G) TTs are not attached to the substratum as confirmed by applying negative or positive pressure through a broken patch pipette. The TT2 was formed by the rear lamellipodium of the cell-1 attached to the rear lamellipodium of cell-2 (encircled).

The primary culture of LSCC cells was prepared from part of the tumor tissue sample, while the second part was used for a histological examination, which revealed the typical properties of LSCC (**Figs. 1A and 1a**) described in the figure legend. LSCC cells were used in experiments between passages 5 to 15 (**Fig. 1B**) when they manifested high proliferative properties (**Fig. 1C**) and mobility (19.4±2.8 µm/h; n = 20), and low membrane potential (−13.7±1.2 mV; n = 40). We found that LSCC cells in the culture formed numerous intercellular tunneling tubes of various width and length (**Fig. 1B**). The thickest (up to 5 µm at the most narrow site) and the longest (up to 1 mm) TTs that could be seen under low magnification (indicated by yellow arrows in **Fig. 1B**) resemble EPBs described by Zani [18]. Due to their microscale width, it is more correct to designate them not TNTs but simply TTs (according to the International Organization of Standardization, definition for “nanoscale” is the size ranging from approximately 1 to 100 nm and for “nano-object” is a material with one, two, or three external dimensions at the nanoscale). LSCC cells also form much thinner (<1 µm) and shorter (up to 100 µm) TTs resembling those first characterized by Rustom and colleagues [9].

It has previously been demonstrated that F-actin and α-tubulin are involved in the formation of TNTs or epithelial bridges (reviewed in refs. [10], [17]). Indeed, the preincubation of LSCC cells possessing the already developed network of TTs for 24 h with colchicine (10 µM), an inhibitor of microtubule polymerization, resulted in a complete loss of TTs (**Fig. 1D**). The inhibitors of actin polymerization, such as cytochalasin B, inhibit TNT formation but weakly affect the already existing TNTs [28]. Similar results we obtained in freshly seeded LSCC cells, which had not developed the network of TTs yet, preincubated with latrunculin A (20 nM), an inhibitor of actin polymerization (not shown).

A specific property of EPBs and TNTs is that they do not attach to the substratum [11], [17]. We demonstrated that TTs formed by LSCC cells were unattached to the substratum by applying positive or negative pressure to the specific TT through a broken patch electrode (**Figs. 1E–1G**). However, the leading edges of lamellipodium extensions were usually attached to the substratum and participated in cell motility and TT formation.

### Modes of TT Formation between LSCC Cells *In vitro*

The analysis of time-lapse imaging allowed us to distinguish the following modes of TT formation: 1) TTs formed during cell division and subsequent dislodgment (TT1); 2) TTs formed by the extensions of rear lamellipodia attached to the remote cell (TT2); 3) TTs formed by the extensions of secondary lamellipodia outgrowing from leading or rear lamellipodia (TT3); 4) TTs formed by the intersection of rear or secondary lamellipodia (TT4); 5) TTs formed by filopodium-like extensions or protrusions (TT5). Thus, for simplification in this paper, TTs will be indexed from 1 to 5 depending on the mode of their formation. We examined the incidence of TT(1–5)s on the 1.2-cm diameter coverslip that contained 3564 fixed immunostained LSCC cells (∼3200 cells per cm^2^) and identified 136 different TTs, among which the incidence of TT1, TT2, TT3, TT4, and TT5 was 18%, 29%, 14%, 11% and 27%, respectively. Similar results were obtained from other 2 coverslips.

#### TT1

**Figure 2:**
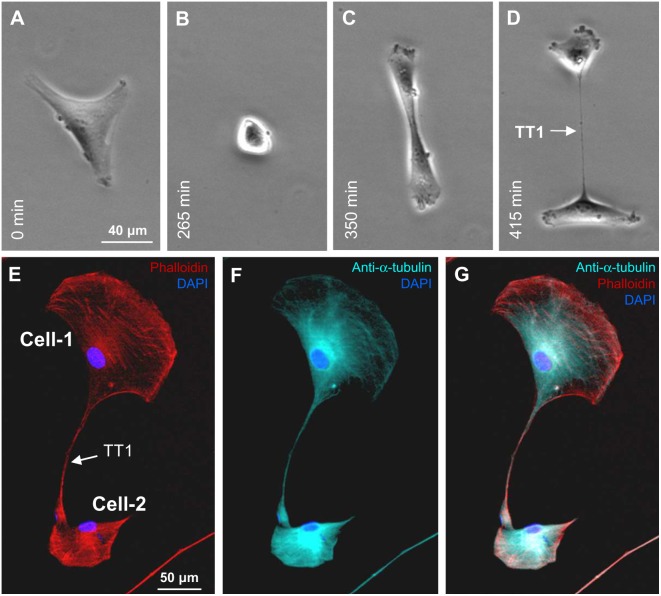
Formation of TT1s between LSCC cells. (A–D) TT1s form in the process of cell division and successive dislodgment. (E–G) TT1s contain both F-actin and α-tubulin.

The typical steps of TT1 formation are shown in **Fig. 2A–2D**. A mature LSCC cell rounded up (A) entering mitosis (B) and divided into 2 cells (C), preserving the cell-to-cell communication during their dislodgment. TT1s were the longest and thickest TTs, up to 1 mm in length and 5 µm in width (D). It has been shown that epithelial bridges contain both F-actin and microtubules [18], while TNTs contain either only F-actin [9] or both F-actin and microtubules [29]. **Figs. 2E–2G** display that TT1s contained both F-actin and microtubules, which were stained with phalloidin and anti-α-tubulin, respectively, suggesting that these TTs might participate in the bidirectional transport of materials [30].

#### TT2

**Figure 3:**
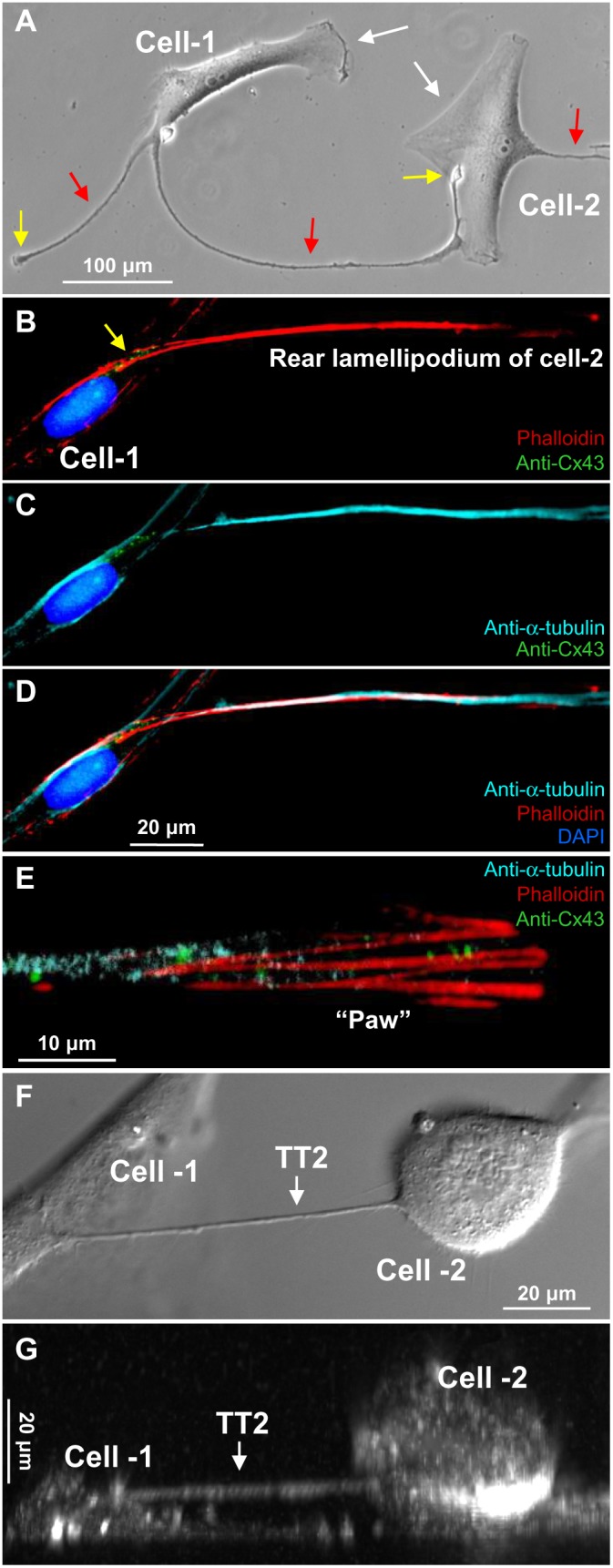
Formation of TT2s between LSCC cells. (A) The leading lamellipodia of cell-1 and cell-2 indicated by white arrows are involved in cell movement. Rear lamellipodia are indicated by red arrows, and one of them outgrowing from the cell-1 forms the TT2 with the cell-2. (B–D) TT2s contain both F-actin and α-tubulin. (E) Crawling endings (“paws”, indicated by yellow arrows in A and B) of the rear and secondary lamellipodia, in addition to F-actin and α-tubulin, contain Cx43 hemichannel clusters. (F–G) Top view (DIC image) and Z-X reconstruction showing the TT2 raised above the substratum.

The formation of TT2 is shown in **Fig. 3A**. The cell-1 had 2 rear lamellipodia (red arrows), and one of them formed the TT2 with the cell-2. Like TT1s, TT2s contained both F-actin and α-tubulin (**Figs. 3B–3D**). The crawling endings lamellipodia (yellow arrows in **Figs. 3A and 3B**), which we call “paws,” in addition to F-actin and α-tubulin threads usually contained Cx43 hemichannel clusters (**Fig. 3E**), which can be utilized to form GJs when the “paw” comes into contact with a remote cell. The Z-X reconstruction showed that TT2s were often raised above the substratum when stretched during cell movement in opposite directions or when cell(s) round up before division (**Figs. 3F and 3G**). The formation of the TT2 is demonstrated in **Movie S1**, where a large rear lamellipodum reached the remote cell, established the TT2 connection with it, and later retracted preserving the connection through newly formed TT5s.

#### TT3

**Figure 4:**
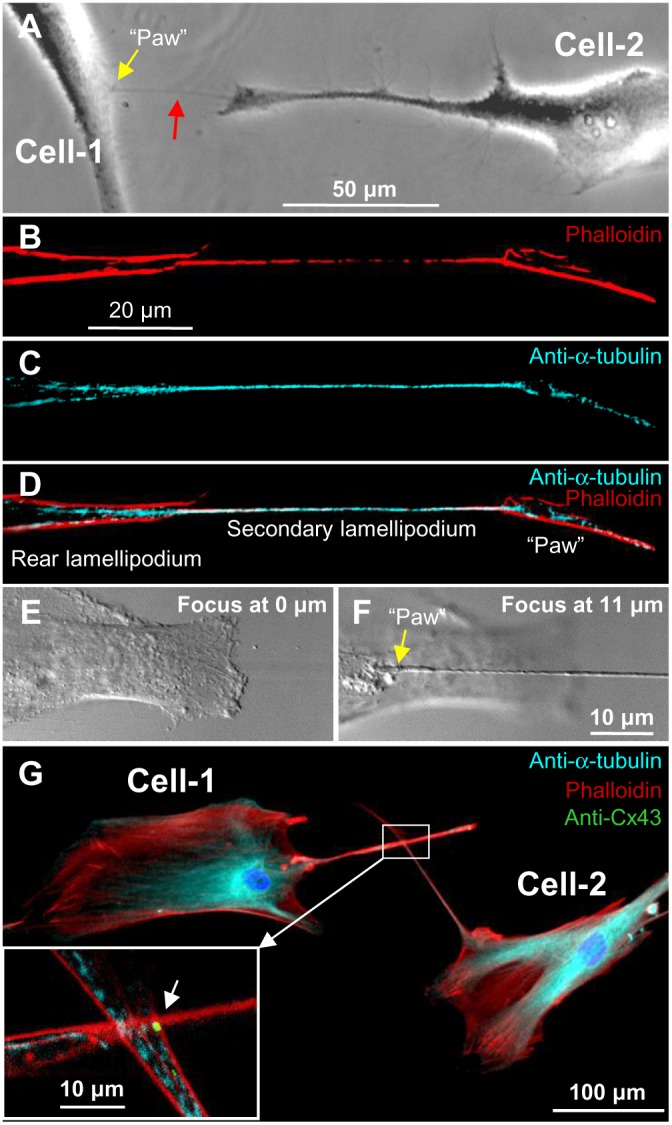
Formation of TT3s between LSCC cells. (A) The secondary lamellipodium (red arrow) outgrowing from the rear lamellipodium of the cell-2 forms TT3 with the cell-1. (B–D) TT3s contain both F-actin and α-tubulin. (E–F) The TT3 between the cell-1 and the cell-2 was found raised 11 µm above the substratum (DIC image). (G) TT4s form between the intersecting rear or secondary lamellipodia establishing functional Cx43 GJs (the arrow in the inset) as confirmed by patch-clamp measurements.

The rear or leading lamellipodia often grew the secondary lamellipodia, which formed the TT3 with the remote cells (**Fig. 4A**, red arrow). TT3s also contained both F-actin and α-tubulin (**Figs. 4B–4D**) and could be seen raised above the substratum (**Figs. 4E and 4F**). The length of TT2s and TT3s measured up to 500 µm and 300 µm, respectively, while their diameter at the most narrow site was about 1–3 µm and 0.5–2 µm, respectively (summary is presented in **Table 1**).

**Table 1:** Summary of TT ultrastructural, electrical, and permeability properties.

Mode	Presence of GJs	L (µm)	d_min_ (µm)	g_T_ (nS)	P_T_×10^−11^ (cm^3^/s)					Transport of mitochondria	Specific marker
					AF350	AF488/3000	LY	DAPI	siRNA/AF488		
TT1	−	380±54 (23)	2.4±0.2 (23)	5.3±1.0 (23)	6.04±1.91 (7)	0.73±0.41 (4)	2.52±1.37 (7)	+(4)	0.34±0.07 (4)	+	F-actin, α-tubulin
TT2	Cx43	174±12 (27)	2.1±0.2 (27)	6.8±0.9 (27)	0.85±0.24 (7)	−	0.16±0.06 (6)	+(5)	0.05±0.01 (6)	+	F-actin, α-tubulin
TT3	Cx43	125±20^#^ (15)	1.2±0.1 (15)	2.6±0.5 (15)	0.24±0.11 (5)	−	0.047±0.015 (5)	+(4)	nd	+	F-actin, α-tubulin
TT4	Cx43	321±46* (11)	1.9±0.2 (11)	3.2±0.7 (11)	0.37±0.16 (4)	−	0.11±0.04 (4)	+(4)	nd	+	F-actin, α-tubulin
TT5	Cx43	44±6 (14)	<0.3^$^ (12)	0.56±0.06 (12)	0.09±0.03 (5)	−	0.027±0.007 (8)	+(4)	nd	−	F-actin

+indicates presence/permeable; – absence/; *sum of two TT lengths measured from the cell body to intersection; ^#^if the secondary lamellipodium was outgrowing from the rear lamellipodium, L was indicated as the length of a secondary one; ^$^diameter of the thinnest TT5s could not be measured precisely by conventional optical microscopy; number of experiments is indicated in parenthesis; nd - no data.

#### TT4

Due to high mobility of LSCC cells, their rear or secondary lamellipodia extensions can intersect forming TT4s (**Fig. 4G**) and establishing functional Cx43 GJs (arrow in the inset) as confirmed by patch-clamp measurements. Such connections can also be established when one of these spindling extensions reach and anchor with its crawling “paw” to the lamellipodium of the second cell.

#### TT5

**Figure 5:**
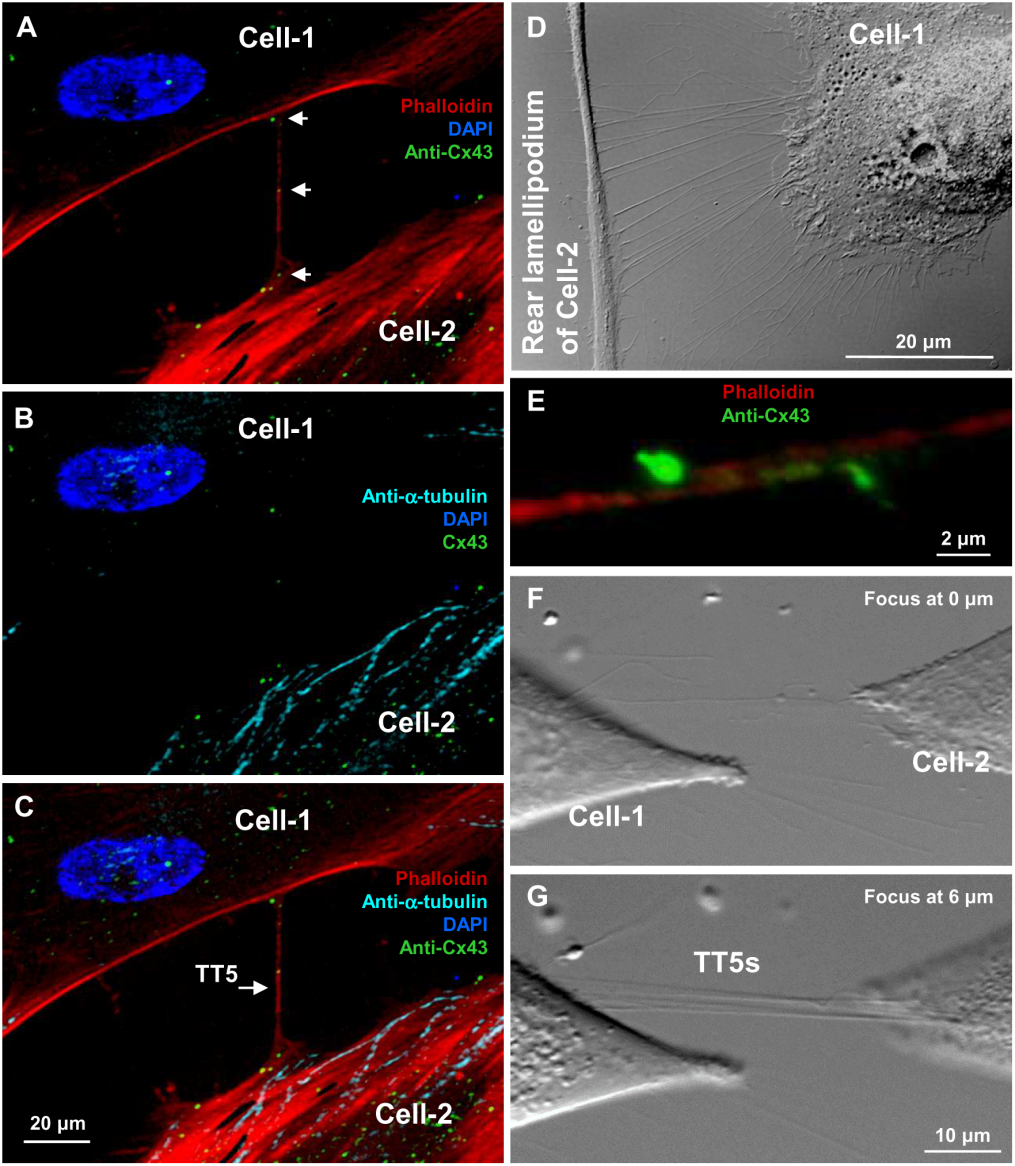
Formation of TT5s between LSCC cells. (A–C) TT5s contain F-actin but not α-tubulin. Cx43 hemichannel clusters, which can be seen inside and/or on the TT5 surface, form functional GJs at the cell border. (D) The multiple TT5s are formed between the cell-1 and the rear lamellipodium of the cell-2 (DIC image). (E) TT5s can be formed by protrusions from apposing cells, which come into contact and form functional Cx43 GJs (green). (F and G) Several TT5s between the cell-1 and the cell-2 were found raised ∼6 µm above the substratum (DIC image).

The thinnest and shortest TTs, i.e. TT5s, formed between LSCC cells when cells first came into contact and then moved apart (**Fig. 5A–5C**) or when filopodium-like protrusions (single or multiple) from one cell connected to another cell (**Fig. 5D**) presumably by forming anchoring junctions containing N-cadherin and β-catenin [31]. Interestingly, two protrusions from the opposing cells can also find each other and form TT5s establishing functional GJs between their tips, where Cx43 accumulation can be seen (**Fig. 5E**). TT5s were also identified as not touching the substratum (**Figs. 5F and 5G**).

TT5s differed structurally from TT(1–4)s in that they did not contain microtubules (**Figs. 5A–5C**). In most cases, TT5s formed Cx43 GJs at the cell border (green dots indicated by white arrows in **Fig. 5A**), and their functionality was confirmed by a dual whole-cell patch-clamp measurement of voltage gating typical of GJs. In addition, cytoplasmic or membranous Cx43 hemichannel clusters can be seen on the surface of both cells and in the middle of TT5.

In our experiments, about 20% (6 out of 32) of TT5s did not couple the cells electrically for 3 possible reasons: 1) TT5s were close-ended, probably involved only in cargo transport on the surface or in the active transport of intracellular materials; 2) GJ-dependent electrical coupling was not established yet in the process of *de novo* formation of TT; 3) GJ-dependent electrical coupling was already lost due to cell separation.

The total conductance of a TT depends on its geometry and the presence or absence of GJs at the contact of a TT with the remote cell. TT length and external diameter can be measured quite precisely; however, the limiting factor of TT conductance is not its external but internal diameter, which can be difficult to estimate.

Cx43, Cx30, and Cx26 have been identified in the laryngeal epithelium and showed no alteration in expression during carcinogenesis [32]. Staining with specific antibodies revealed that indeed LSCC cells in culture (**Figs. S1A–S1C**) and in tissue (**Figs. S1D–S1F**) expressed Cx43, Cx30, and Cx26; however, while in the cell culture, Cx43 GJs could be seen between 2 abutted cells (∼1 µm green dots between cell-2 and cell-3 in **Fig. S1A**), Cx26 and Cx30 could be found rather as membranous and/or intracellular clusters of hemichannels (**Figs. S1B and S1C**). To determine whether these 3 types of Cxs participate in TT-mediated intercellular communication between LSCC cells, we measured specific single channel conductances using the patch-clamp technique (see below).

### Permeability and Electrical Properties of TTs

**Figure 6:**
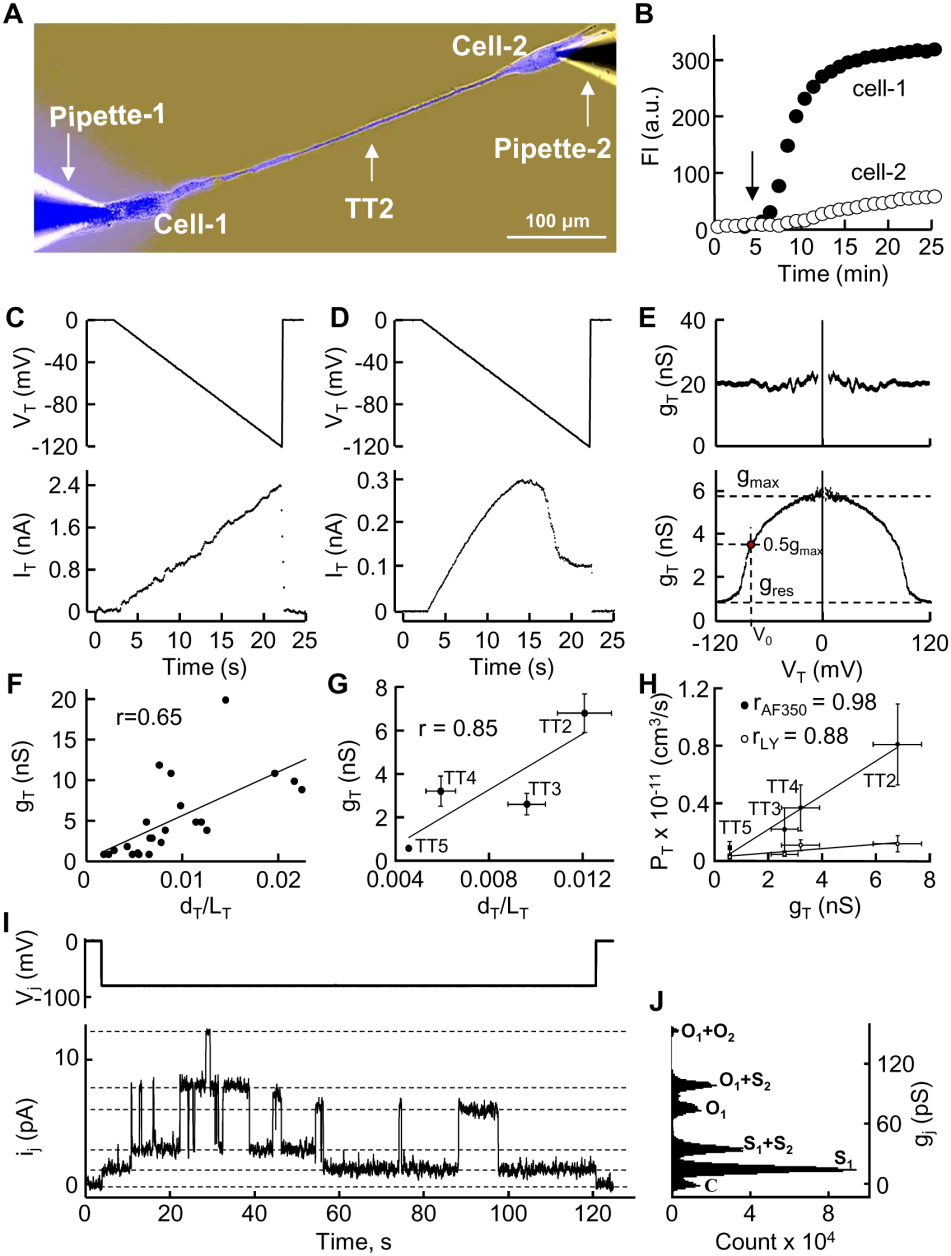
Permeability and electrical properties of TTs. (A) AF350 dye introduced into the cell-1 through the patch pipette-1 diffuses via the TT to the cell-2. (B) Kinetics of dye accumulation in the cell-1 and the cell-2 after opening of patch-1 (indicated by arrow). (C–D) Electrical properties of TT were measured by opening patch-2 at the end of the experiment, applying the voltage ramp of negative polarity from 0 to −120 mV (upper panels), and measuring junctional current in the cell-1 (lower panels). I_T_ responses in (C) and (D) are typical of TTs without and with GJs, respectively. (E) g_T_/V_T_ dependence is calculated from I_T_ response to V_T_ ramp (shown in C and D) and presented with its symmetric counterpart. The absence or presence of V_T_ gating indicates that the TT does not contain or contains GJs (upper and lower panel, respectively). (F) Correlation between TT1 conductance and geometry. (G) Correlation between TT(2–5)s conductance and geometry. TT5s thinner than 200 nm, the diameter of which cannot be measured by conventional microscopy, were not included into statistical analysis. (H) Correlation between TT permeability and electrical conductance. (I) Recording of single-channel current i_j_ (lower trace) in response to 2-min −80 mV voltage pulse V_j_ (upper panel). (J) Single channel substate (S) and open state (O) conductance γ_j_ was estimated by an all-point histogram fitted by the Gaussian function (pClamp 10 software). Two functioning channels can be recognized with respective substates and open states typical of Cx43 channels (more details are provided in the main text).

One of the goals of this study was to determine the permeability of TTs formed by different modes to dyes that differ in molecular mass and charge. To measure TT permeability, the pipette-1 filled with the dye was attached to the cell-1 and the pipette without the dye was attached to the cell-2 (**Fig. 6A**). After opening patch-1, the dye diffused to the cell-1 followed by dye transfer through the TT to the cell-2. Depending on TT length, there was a delay for the dye to reach the cell-2 caused by dye spread along the TT. Typically, fluorescence intensity in the cell-1 reached steady state during ∼10–20 min, but this time was longer for the cell-2 (**Fig. 6B**). The total TT permeability, P_T_, was evaluated using equation 3, which accounted for changes in fluorescence intensity in the cell-1 (FI1) and the cell-2 (FI2). It has previously been reported that GJs allow the passage of molecules smaller than 1.2 kDa [33]. We checked if TTs not containing GJs permitted the passage of larger molecules. Indeed, TT1s were permeable to at least 3-kDa molecules, such as AF488/3000. All TT(1–5)s were permeable to other used dyes of lower molecular weight (AF350, LY, and DAPI), and permeability strongly depended on the net charge of the dye. For instance, there was only a 3-fold difference in the permeability of the TT1 to LY (valence −2) and AF488/3000 (valence −1), while their molecular mass differed by 7 fold, 443 and ∼3000, respectively. Moreover, although the molecular mass of AF350 (valence −1) and LY was comparable, their permeability magnitudes in TT(1–5)s differed several times. The permeability properties of TTs formed by different modes are summarized in **Table 1**. Since positively charged dyes bind to nucleotides, DAPI was not quantitatively assessed, and **Table 1** only indicates that all TT(1–5)s were permeable to it.

At the end of the dye transfer measurement, if satisfactory conditions of voltage clamping in the cell-1 persisted, the patch in the cell-2 was opened to measure electrical conductance of the TT, g_T_, in a dual whole-cell voltage-clamp mode. Additional experiments were carried out to quantify only the electrical properties of TT(1–5)s without the measurement of dyes transfer. TT1s typically were open-ended, i.e. they did not possess the properties of voltage gating characteristic of GJs. This was demonstrated by measuring the g_T_/V_T_ dependence of the TT and/or by application of octanol, a GJ blocker (not shown). **Fig. 6E** (upper panel) displays the g_T_/V_T_ plot obtained by measuring the I_T_ response (**Fig. 6C**, lower panel) to the voltage ramp of negative polarity from 0 to −120 mV (**Fig. 6C**, upper panel) with its symmetric counterpart. The absence of voltage gating suggests that the TT did not contain the GJ. TTs containing GJs demonstrated voltage-gating properties. Such a typical g_T_/V_T_ plot (**Fig. 6E** **,** lower panel) was obtained by the same procedure, and the I_T_ response to the voltage ramp is shown in **Fig. 6D**.

TT conductances correlated with their geometry assuming that g_T_ depends directly on TT width and inversely on TT length. In case of TT1, the correlation was moderate (r = 0.65) and we believe that the correlation would improve if an internal diameter of TT1s were estimated instead of external one (**Fig. 6F** **;** data are taken from **Table 1**). The mean conductances of TT(2–5)s correlated well with their geometry (**Fig. 6G**); however, it is necessary to note that in single experiments, in the TTs containing GJs, g_T_ was unrelated to TT geometry because neither TT length nor width and nor size of a “paw” determine the size of junctional plaque (JP) as well as the total number of channels and number of functional channels in it, the same as in case of abutted cells, the larger contact area does not guarantee a larger size or number of JPs, and higher intercellular conductance. On the other hand, the mean permeabilities of TT(2–5)s to fluorescent dyes such as AF350 and LY correlated well with their mean conductances (**Fig. 6H**). In contrast, the conductance of TT1s corresponded to higher permeability pointing to that GJs provided a higher selectivity filter than TTs themselves.

**Fig. S1** depicts that LSCC cells may express Cx26, Cx30, and Cx43; however, the expression of these Cxs does not mean that they all form GJs. It is known from other studies that single-channel conductances (open state/residual state) of human Cx26, Cx30, and Cx43 are 115–150/30, 160/27, and 90–110/30 pS, respectively [34], [35]. To check if all these conductances can be detected in TT(2–5)-mediated junctions, we employed the dual whole-cell patch-clamp technique. Since junctions usually contained multiple functional channels (mean conductance between abutted cells was 33.6±6.2 nS (n = 15), and g_T_ between cells connected through TT(1–5)s is shown in **Table 1**), to measure single-channel conductances, we used 0.5 mM octanol, which strongly reduced electrical coupling between cells and allowed to record single-channel openings. In all experiments in which we succeeded to record single-channel openings at V_j_ = −80 mV (**Fig. 6I** **, upper panel**), voltage which closes both fast and slow gates of the channels [8], we detected only single-channel currents (**Fig. 6I** **, lower panel**) and conductances (**Fig. 6J**) typical of Cx43 GJs. The mean single-channel conductances of open and residual states, respectively, were 94±6 pS and 32±3 pS (n = 11) for abutted cells and 93±6 pS and 33±3 pS (n = 9) for cells connected through TTs containing GJs.

### Cargo Transport through the TTs

**Figure 7:**
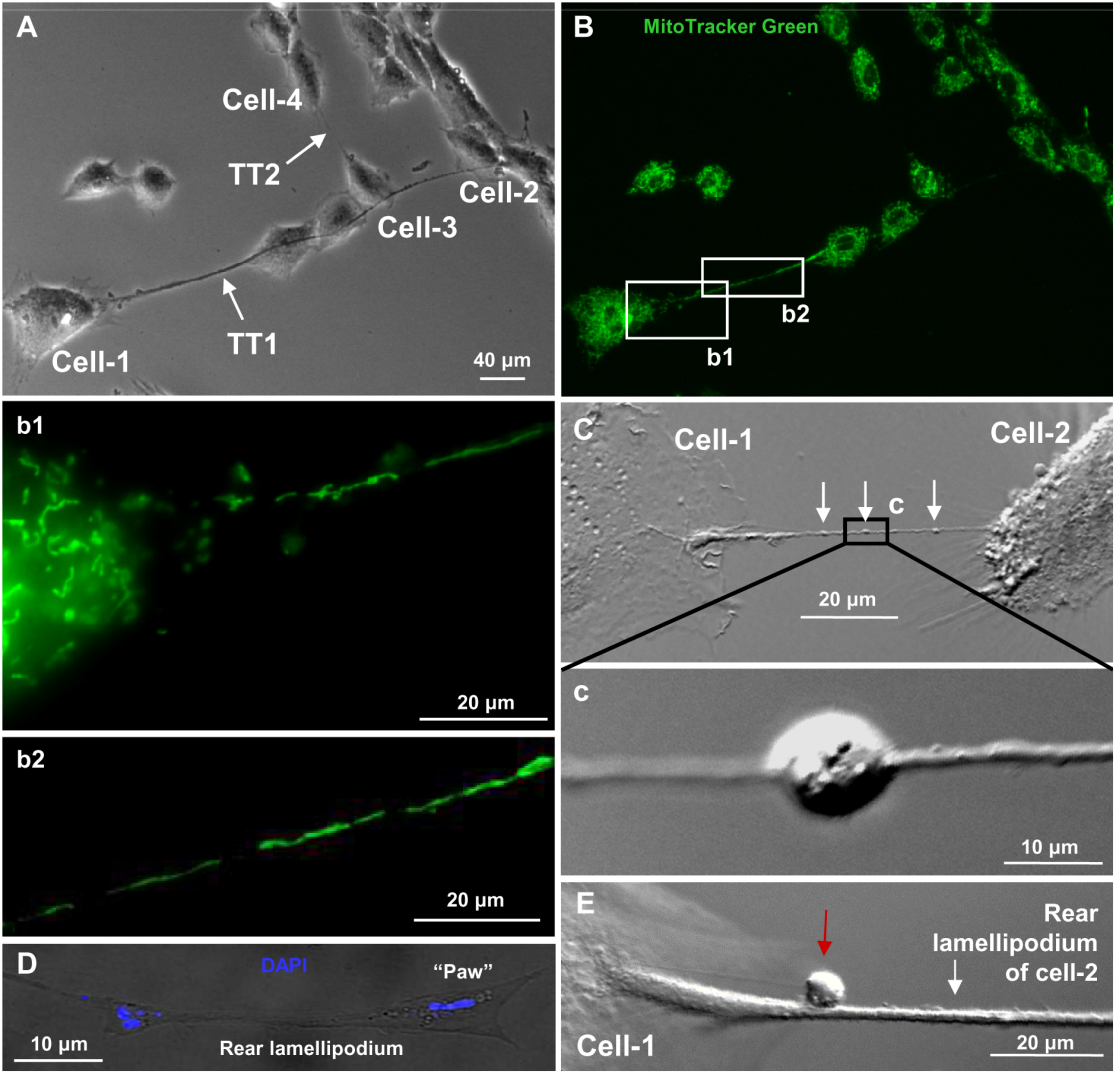
TTs between live LSCC cells in the culture contain mitochondria. (A) A phase-contrast image shows the TT1 connecting the cell-1 and the cell-2, and the TT2 connecting the cell-3 and the cell-4 (arrows). (B) A dense network of mitochondria stained with MitoTracker Green is present in all cell bodies and also in the TT1 and the TT2 (insets b1 and b2). (C and c) TTs can be involved in cargo transport (DIC image). (D) The TT2 contains small DAPI-positive vesicles. (E) Cargoes can be transported along an outer surface of TTs (red arrow) (DIC image).

The striking feature of TNTs is that they can transfer not only small molecules such as Ca^2+^, IP_3_, glutamate, glutathione, ADP, and ATP but also lysosomal and intracellular vesicles (early endosomes, endoplasmic reticulum, Golgi) and even cellular organelles, such as mitochondria (reviewed in ref. [11]). To examine whether LSCC cells contain and are capable of transferring mitochondria, we used MitoTracker Green, a dye specific to mitochondria. **Fig. 7A** shows a ∼200 µm TT1 passing above 3 other cells and connecting the cell-1 and the cell-2, as well as a shorter TT2 connecting the cell-3 and the cell-4. All cell bodies in **Fig. 7B** demonstrate a dense network of mitochondria, which are also present and can move inside TTs (see insets **b1** and **b2** of **Fig. 7B** **,** and **Movie S3**). Smaller TTs, such as TT2s or TT3s, also contain mitochondria, which can be seen under higher magnification (not shown); however, we failed to detect mitochondria in TT5s.

TTs can be involved in other cargo transport (white arrows in **Fig. 7C, 7c** and **Movie S2**) by a mechanism presumably involving actin-binding motor myosin-Va, which has been shown to partially co-localize with endocytic organelles [36]. We observed cargo movement either inside the TTs (**Fig. 7c**) or along their outer surface (**Fig. 7E** **,** red arrow).

**Figure 8:**
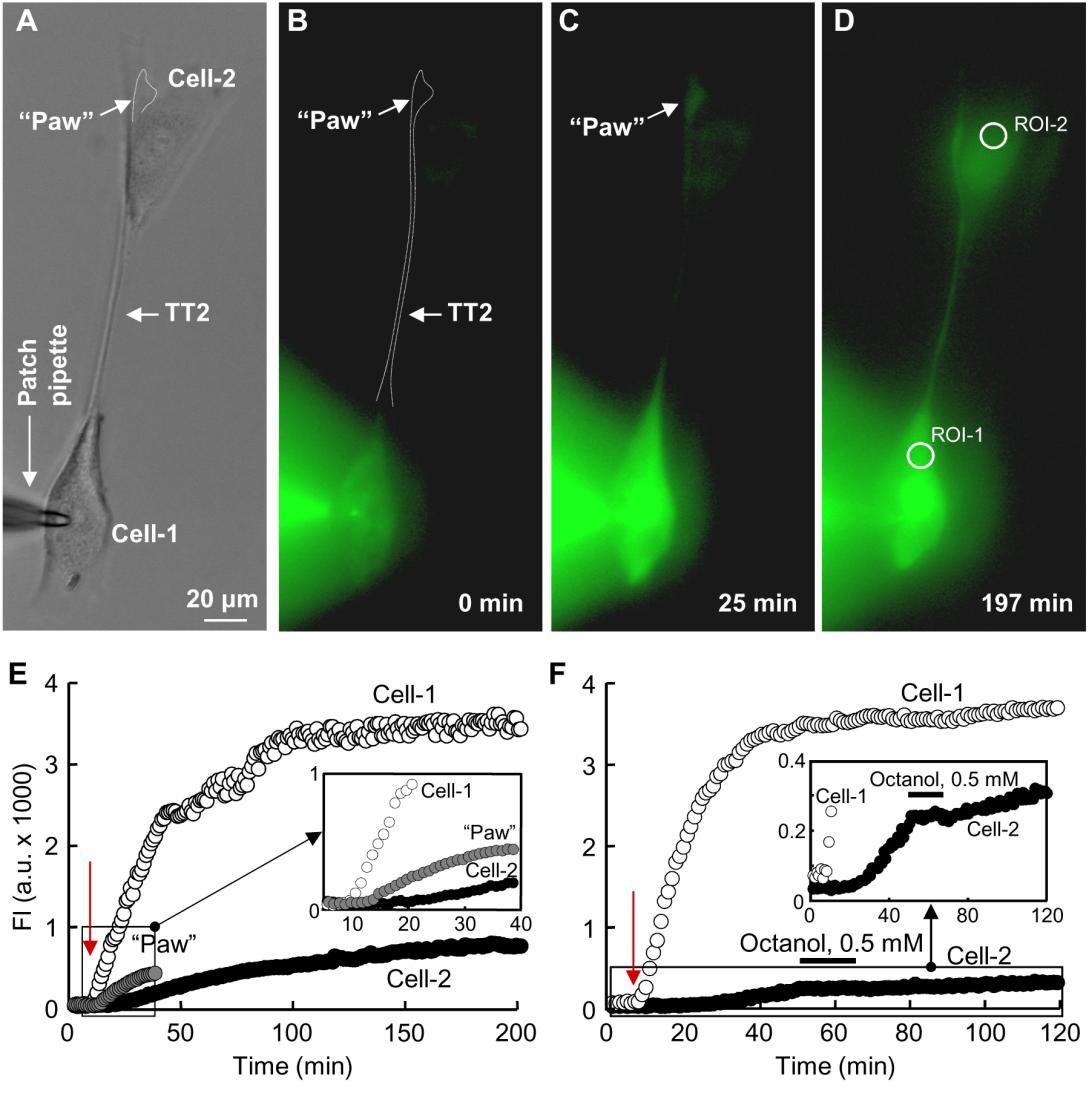
Permeability of TTs between LSCC cells to siRNA/AF488 in the culture. (A) A phase-contrast image shows the TT2 connecting the cell-1 and the cell-2. SiRNA/AF488 (2 µm) introduced into the patch pipette enters the cell-1 after patch opening (B), rapidly diffuses along the TT2 to the “paw,” and then slowly accumulates through the GJ in the cell-2 (C). (E and inset) Kinetics of siRNA/AF488 accumulation in the cell-1, “paw,” and cell-2. (F and inset) SiRNA transfer through the TT2 containing GJs was confirmed by application of octanol (0.5 mM), which reversibly arrested siRNA/AF488 accumulation in the cell-2. A red arrow indicates the patch opening time in the cell-1.

Interestingly, in TT(1–4)s, we observed extremely small, 1–2 µm in diameter, DAPI-stained vesicles (**Fig. 7D**), the fluorescence of which clearly differed from background autofluorescence of intracellular components such as aromatic amino acids, lipopigments, and pyridine and flavin coenzymes [37]. These vesicles resemble DAPI-stained mitochondrial DNA detected in human osteosarcoma cell bodies [38]; however, the diameter of nucleoids in which mitochondrial DNA is localized in the tubular mitochondria is ∼99 nm [39], i.e., much smaller than that of vesicles shown in **Fig. 7D**. Even agglomerates of several nucleoids do not exceed 300 nm in diameter. We assume that these vesicles may contain miRNA or siRNA, suggesting a possible transfer of nuclear materials through TTs. To verify if double-stranded siRNA can be transported from cell to cell via TTs containing and not containing GJs, we performed fluorescence imaging experiments using control double-stranded siRNA conjugated with Alexa Fluor-488 (siRNA/AF488). **Fig. 8A** demonstrates a bright field image of 2 LSCC cells connected with the TT2. siRNA/AF488 (2 µM) added into the patch pipette, after the patch opening, entered the cell-1, diffused along the TT2 to its “paw” and then slowly accumulated in the cell-2 (**Fig. 8B–8D** and **Movie S4**). The kinetics of dye accumulation in the cells is shown in **Fig. 8E**. To confirm that siRNA/AF488 is able to permeate through TTs, containing GJs, we performed similar experiments where octanol (0.5 mM; 15 min) was applied to close GJ channels during the process of dye accumulation in the cell-2. Under such conditions, octanol arrested siRNA/AF488 accumulation in the cell-2, which was reinitiated after the washout of octanol (**Fig. 8F**). Due to high molecular weight and net negative charge, the accumulation of siRNA/AF488 was much slower than that of other fluorescent dyes used. P_T_ of TT1 and TT2 was 3.4±0.7×10^−12 ^cm^3^/s (n = 4) and 5.0±1.1×10^−13 ^cm^3^/s (n = 6), respectively (see summary in **Table 1**).

### TTs in the LSCC Tissue

**Figure 9:**
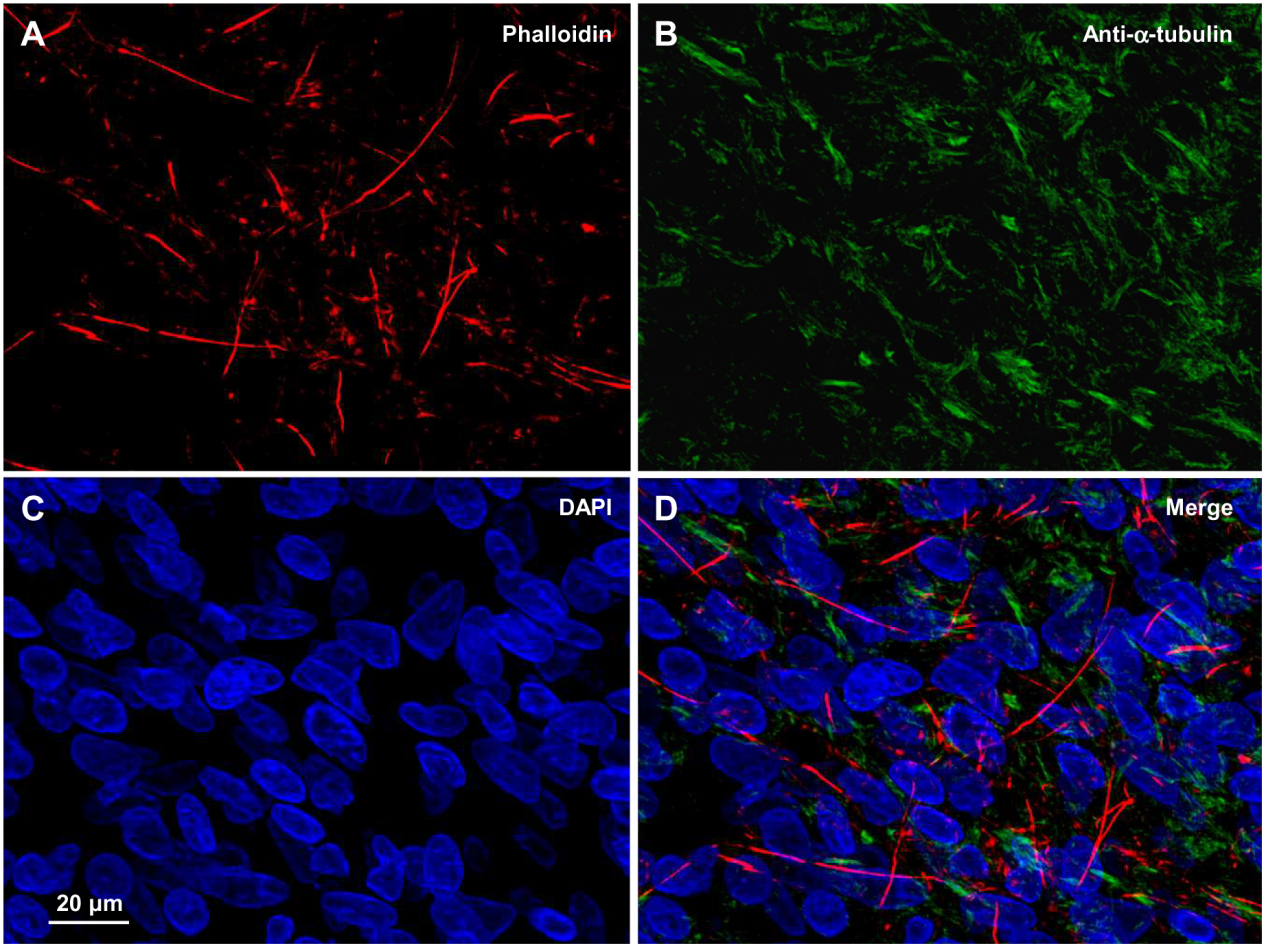
TT5-like tunneling tubes in the LSCC tissue samples. (A–D) TTs containing F-actin but not α-tubulin.

To date, only few publications on TNT existence in tissues have been published. TTs were identified in the mouse corneal stroma [19], neonatal rabbit during nephron induction between mesenchymal and epithelial stem cells [40], specimens of human pleural mesothelioma and adenocarcinoma [20], and between migrating cells of human ovarian cancer explant cultures [21]. To determine whether TTs exist not only between LSCC cells in the culture but also in solid tumors, we microsectioned and analyzed samples taken from 6 patients with LSCC (from one of these samples, a primary cell culture was developed and characterized, as shown above). Since there are no other specific markers of TTs, the sections were stained with phalloidin and anti-α-tubulin. As in the cell culture, we identified 2 types of TTs. The TTs of the first type were up to 1 µm in width and up to ∼100 µm in length with solely F-actin threads spanning the entire length of TTs (**Fig. 9** and **Movie S5**). Such TTs could be attributed to TT5s found in the cell culture.

**Figure 10:**
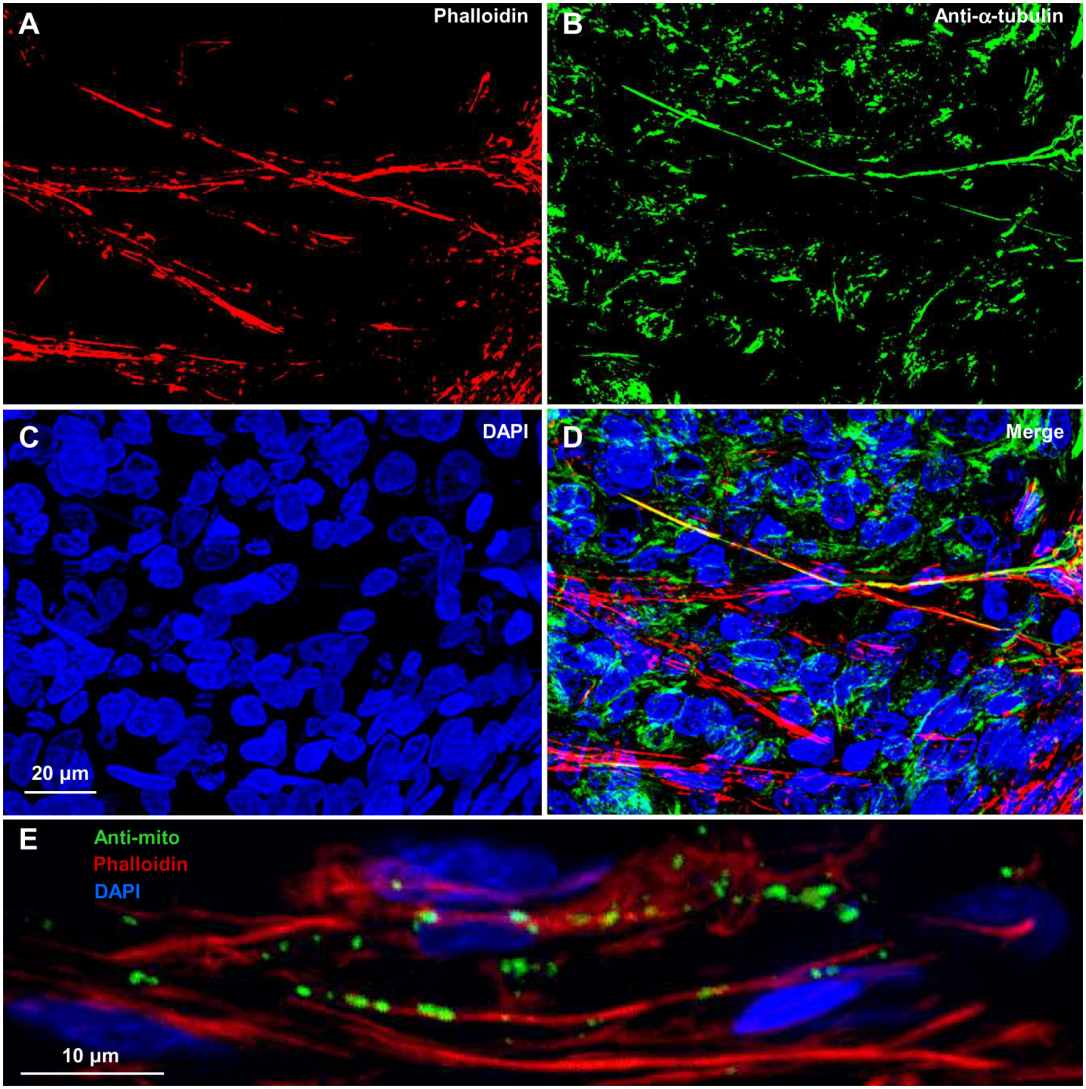
TT(1–4)-like tunneling tubes in the LSCC tissue samples. (A–D) TTs containing F-actin and α-tubulin. (E) TTs containing mitochondria co-localizing with F-actin.

The TTs of the second type were longer (up to 300 µm) and thicker (∼2–3 µm) with co-localizing both cytoskeleton components, F-actin and α-tubulin (**Figs. 10A–10D**). Moreover, in these TTs, mitochondria could be seen co-localizing with F-actin (**Fig. 10E** **)**. Such TTs could be attributed to TT(1–4)s found in the cell culture. Unfortunately, in both cases, it was problematic to quantitatively assess the geometry of TTs as we did in the cell culture because 25-µm tissue sections rarely contained entire not damaged TTs.

## Discussion

In the current study, we have demonstrated for the first time in the primary cell culture prepared from human LSCC samples that LSCC cells were able to communicate with each other over long distances through membranous TTs. To support the idea that the phenomenon of TTs is typical not only of cells *in vitro*, we have examined the microsections of LSCC tissue samples and identified intercellular structures similar to those found in the cell culture. We have not only identified 5 modes of TT formation in the culture but also provided the quantitative assessment of TT electrical properties and permeability to fluorescent dyes of different molecular weight and charge (**Table 1**). Also, we have shown that mitochondria residing inside the TTs containing α-tubulin are mobile and presumably can transit through TTs from one LSCC cell to another as it has been demonstrated in other cell types [21], [41]. TT1s but not TT(2–5)s containing GJs are possible candidates for such transition. TT1s form during mitotic cell division, which ends in daughter cell separation, known as cytokinesis. This mechanism involves the formation of a TT1-like intercellular bridge, and the final step of cytokinesis is abscission. Intercellular bridges are relatively short structures from several microns to several tens of microns in length and 1–3 µm in thickness depending on the cell type [42], while in our study, we have identified extremely long, up to 1 mm TT1s pointing to that cancer cell separation might be abnormal, and preservation of such long connections, which allow fast trafficking of mitochondria, could facilitate cancer cell invasion and progression.

It has been shown before that the laryngeal epithelium in addition to Cx43 expresses Cx26 and Cx30 with no alteration in expression during carcinogenesis [32]. Our immunofluorescence measurements confirmed the expression of these 3 types of Cxs in the LSCC tissue and the cell culture; however, we identified only functional Cx43 GJ channels by the patch-clamp technique either between the cells abutted or connected through TT(2–5)s, thus far. Therefore, the role of Cx26 and Cx30 remains to be elucidated. These Cxs may play an important role in the transfer of materials because their single-channel conductances (and presumably pore sizes) are even larger than those of Cx43 channels. Moreover, heterotypic Cx43/Cx26 and Cx43/Cx30 junctions (at least Cx43 and Cx30 have been shown to be compatible [43]) can be important in directed transport because fast gates of Cx26 and Cx30 hemichannels exhibit positive polarity, while Cx43, negative. Due to opposite gating polarities of apposed hemichannels (aHCs) in the GJ, one V_j_ polarity tends to open and the opposite V_j_ polarity tends to close both aHCs, determining the sigmoidal g_T_/V_T_ dependence of heterotypic GJ channels instead of the symmetric bell-shaped one of homotypic junctions (see **Fig. S2**). In many cases, cancerous cells have been shown to have significantly lower resting potential than normal cells or fibroblasts [44]; therefore, for instance, heterotypic Cx43/Cx30 junctions between cancer cells and cancer-associated fibroblasts (CAFs) could be tuned by changes in membrane potential which, in turn, is determined by membranous ion channels and transporters. Reduced membrane potential may be functionally related to mechanisms by which oncogene-bearing cells switch from normal morphogenesis to form tumors, and the modulation of membrane potential has recently been suggested as a novel strategy for tumor normalization [45]. The asymmetry of electrical signal transfer, which can be modulated from unidirectional to bidirectional by small changes of V_m_ in one of the cells, was demonstrated in other heterotypic junctions (reviewed in ref. [43]).

In carcinogenesis, a very important role is played by fibroblasts, which having been transformed to CAFs by paracrine signaling from carcinoma cells, in turn, induce an epithelial-to-mesenchymal transition of carcinoma cells together with which secrete several extracellular matrix-degrading enzymes opening pathways for a tumor cell invasion [46], [47]. In these processes, signaling via TTs may be more effective than paracrine one, in particular if TTs form between cancer cells and fibroblasts or stem cells. Communication between cells via TT5s should mainly maintain homeostasis, while communication between cancer cells and CAFs by TT(2–4)s may fuel cancer cells with high-energy nutrients and even mitochondria (**Fig. 7** and **Movie S3**). This idea is consistent with the recent publication that has demonstrated the preferential transfer of mitochondria from endothelial to cancer cells resulting in chemoresistance [21]. Mitochondria play an important role in tumor progression; however, their role in CAFs and cancer cells is different. A recent finding of Sanchez-Alvarez and colleagues [48] suggests that mitochondrial dysfunction in CAFs promotes tumor growth via ketone production, while their dysfunction in epithelial cancer cells has an opposite effect and inhibits tumor growth.

Tumor microenvironment includes CAFs, macrophages, endothelial cells, etc. interacting not only by soluble cytokines and growth factors but also by the release of cargo-vesicles, such as exosomes or oncosomes, which can carry ATP, proteins, or miRNAs [49]. Vesicular transport through TNTs containing F-actin and α-tubulin was shown to be energy-dependent and to require microtubule molecular motors such as kinesins [30]. Our finding that TTs contain DAPI-stained vesicles and can deliver siRNA/AF488 to the remote cells suggests the existence of a direct pathway for miRNA or siRNA transport between cells, which might be much faster and efficient than pinocytotic/exosomal routes. In this pathway, GJs should not be a limiting factor. Several studies suggested that GJs might be permeable to miRNAs and siRNAs [50]–[53] despite their high molecular mass but due to rod shape morphology allowing their passage through GJs with larger pores [53]. For instance, Valiunas et al. have demonstrated that siRNA can be transported through GJs composed of Cx43 but not Cx26 or Cx32 [51]. Specific gene silencing has therapeutic potential; however, the delivery of exogenous siRNA to the interior of target cells is problematic. This problem could be resolved by using the human mesenchymal stem cell (hMSC)-based siRNA delivery system [53]. These cells are not immunoreactive, exhibit high proliferative potential, and express Cx43.

Having provided the first characterization of long-distance communication between LSCC cells through TTs, we believe that these findings allow a better understanding of cancer cell biology and deserve further investigations focusing on the following: 1) TT-mediated communication between CAFs, hMSCs, and LSCC cells, including siRNA transfer and manipulation of gene expression, and 2) regional differences in the structure of TTs and Cx26, Cx30, and Cx43 expression in LSCC tissues involving tumor parenchyma, stroma, and transitional zone, where the formation of heterotypic GJs may be important for cancer metastasis.

## Supporting Information

Figure S1 **Immunostaining of Cx43, Cx30, and Cx26 in the LSCC cell culture (A–C) and tissue (D–F), respectively.** Connexins are shown in green, F-actin in red, and nucleus in blue pseudo colors.
(TIF)

Figure S2 **Simulation of homotypic and heterotypic gap junction g_j_/V_j_ relationships by the S4SM model** **[54].** (A) Two hemichannels forming a homotypic gap junction are composed of Cx43^−^ with negative gating polarity. (B) Two hemichannels forming a heterotypic gap junction are composed of Cx26^+^ and Cx30^+^, respectively, both gating at positive voltages. (C) Two hemichannels forming a heterotypic gap junction are composed of Cx26^+^ and Cx43^−^, respectively, the first gating at positive and the second at negative voltages. (D) Two hemichannels forming heterotypic gap junction are composed of Cx43^−^ and Cx30^+^, respectively, the first gating at negative and the second at positive voltages. Parameters γ_open_, γ_residual_, V_0_, and A of Cx26, Cx30, and Cx43 GJs are taken from a review article by Gonzalez et al. [34]. ^−^ and ^+^ indicate gating polarity of Cxs.
(TIF)

Table S1 **List of filters used for the visualization of an appropriate fluorescent marker.**
(DOC)

Movie S1 **Formation of TT2 and TT5 between LSCC cells in the culture.**
(AVI)

Movie S2 **Cargo transport along TT2 between LSCC cells in the culture.**
(AVI)

Movie S3 **Movement of mitochondria inside the TT2 between LSCC cells in the culture.** Mitochondria in live cells were labeled with MitoTracker Green.
(AVI)

Movie S4 **SiRNA/AF488 transport through the TT2 between LSCC cells in the culture.** SiRNA/AF488 (2 µM) was loaded into the cell-1 through the patch pipette, diffused along the TT2 to its ending situated on the cell-2, and then slowly accumulated in the cell-2.
(AVI)

Movie S5 **3D picture of the 25-µm LSCC tissue section.** F-actin is stained with phalloidin (red color) and nucleus with DAPI (blue color). While short F-actin fibers may represent an intracellular F-actin network, long ones should be attributed to the intercellular TTs.
(AVI)
