# Supplementary figures and images for: Long-Distance Communication between Laryngeal Carcinoma Cells
Source: PLoS One. 2014 Jun 19;9(6):e99196. doi: 10.1371/journal.pone.0099196 (PMC4063716; doi:10.1371/journal.pone.0099196)

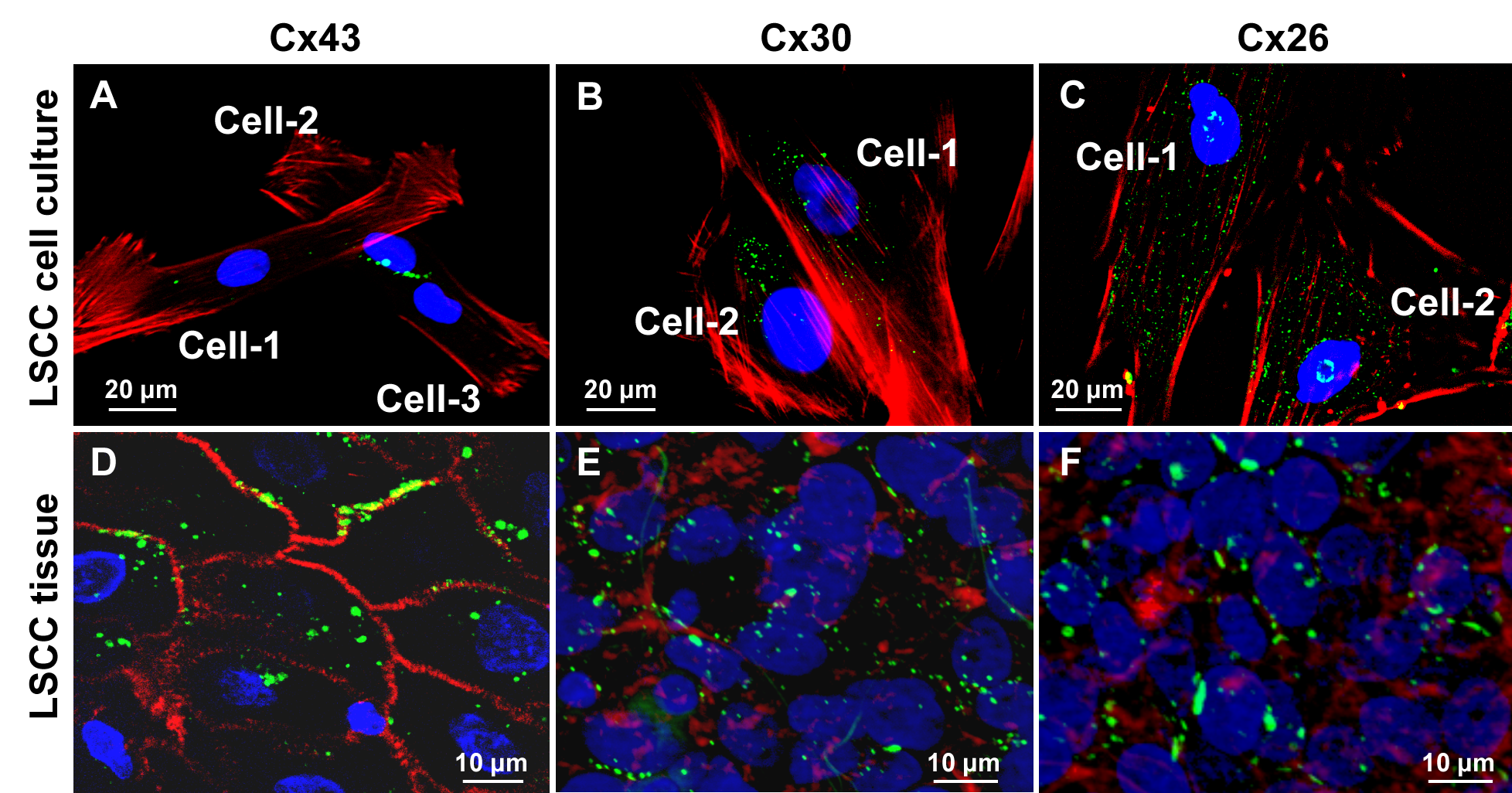

Supplement: Figure S1 — Immunostaining of Cx43, Cx30, and Cx26 in the LSCC cell culture (A–C) and tissue (D–F), respectively. Connexins are shown in green, F-actin in red, and nucleus in blue pseudo colors. (TIF) [file pone.0099196.s001.tif]

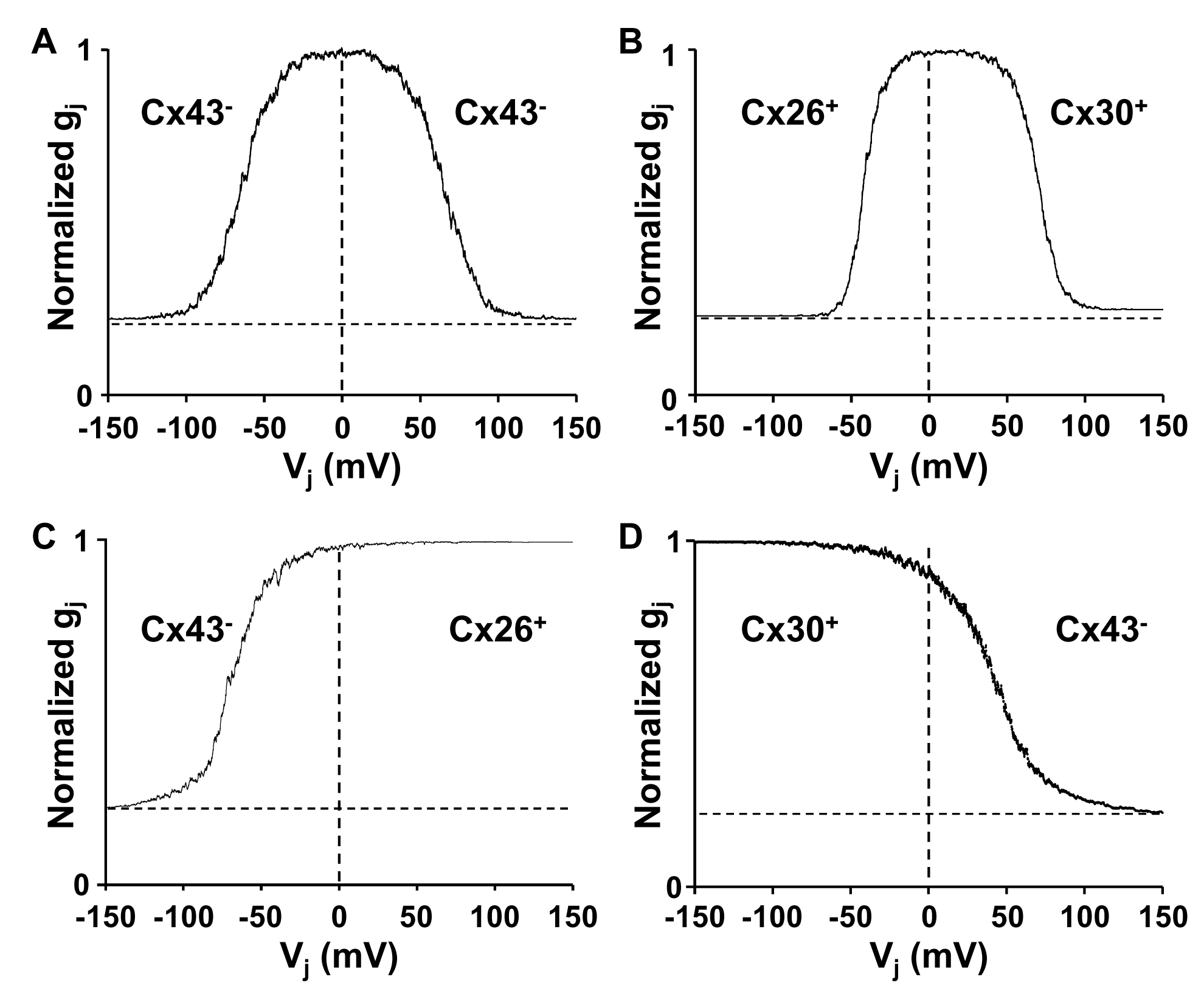

Supplement: Figure S2 — Simulation of homotypic and heterotypic gap junction gj/Vj relationships by the S4SM model [54]. (A) Two hemichannels forming a homotypic gap junction are composed of Cx43− with negative gating polarity. (B) Two hemichannels forming a heterotypic gap junction are composed of Cx26+ and Cx30+, respectively, both gating at positive voltages. (C) Two hemichannels forming a heterotypic gap junction are composed of Cx26+ and Cx43−, respectively, the first gating at positive and the second at negative voltages. (D) Two hemichannels forming heterotypic gap junction are composed of Cx43− and Cx30+, respectively, the first gating at negative and the second at positive voltages. Parameters γopen, γresidual, V0, and A of Cx26, Cx30, and Cx43 GJs are taken from a review article by Gonzalez et al. [34]. − and + indicate gating polarity of Cxs. (TIF) [file pone.0099196.s002.tif]
