# Supplementary material for: Long-Distance Communication between Laryngeal Carcinoma Cells
Source: PLoS One. 2014 Jun 19;9(6):e99196. doi: 10.1371/journal.pone.0099196 (PMC4063716; doi:10.1371/journal.pone.0099196)
Supplement: Table S1 — List of filters used for the visualization of an appropriate fluorescent marker. (DOC) [file pone.0099196.s003.doc]

**Table S1.** The filters used for visualization of an appropriate fluorescent marker.

| **Fluorescent marker** | **Excitation filter** | **Emission filter** |
| --- | --- | --- |
| Alexa Fluor-350 (AF350) | AT350/50x | D470/40m |
| DAPI |
| Lucifer Yellow (LY) | D410/40x | HQ525/50m |
| Alexa Fluor-488/3000 dextran (AF488/3000) | HQ470/40x | HQ525/50m |
| siRNA conjugated with AF488 (siRNA/AF488) |
| Secondary antibodies conjugated with fluorescein isothiocyanate (FITC) |
| MitoTracker Green |
| Alexa Fluor-594 phalloidin | HQ540/40x | HQ600/50m |
| Cyanine-5 (Cy5) | HQ640/20x | HQ700/75m |
